# Extending long-range phasing and haplotype library imputation methods to impute genotypes on sex chromosomes

**DOI:** 10.1186/1297-9686-45-10

**Published:** 2013-04-25

**Authors:** John M Hickey, Andreas Kranis

**Affiliations:** 1 School of Environmental and Rural Science, University of New England, Armidale, Australia; 2 Biometrics and Statistics Unit, Crop Research Informatics Lab, International Maize and Wheat Improvement Center (CIMMYT), 06600, Mexico, DF, Mexico; 3 Aviagen Ltd., Newbridge, Scotland EH28 8SZ, UK

## Abstract

AlphaImpute is a flexible and accurate genotype imputation tool that was originally designed for the imputation of genotypes on autosomal chromosomes. In some species, sex chromosomes comprise a large portion of the genome. For example, chromosome Z represents approximately 8% of the chicken genome and therefore is likely to be important in determining genetic variation in a population. When breeding programs make selection decisions based on genomic information, chromosomes that are not represented on the genotyping platform will not be subject to selection. Therefore imputation algorithms should be able to impute genotypes for all chromosomes. The objective of this research was to extend AlphaImpute so that it could impute genotypes on sex chromosomes. The accuracy of imputation was assessed using different genotyping strategies in a real commercial chicken population. The correlation between true and imputed genotypes was high in all the scenarios and was 0.96 for the most favourable scenario. Overall, the accuracy of imputation of the sex chromosome was slightly lower than that of autosomes for all scenarios considered.

## Findings

AlphaImpute [1] is a tool for imputing genotypes in pedigreed populations that is flexible to the pedigree structure of genotyped animals and works for large datasets. It involves simple phasing rules, long-range phasing and haplotype library imputation [2,3], and segregation analysis [4]. It was initially designed to work for autosomes and therefore did not perform imputation of genotypes on sex chromosomes. When genomic information is used to make the selection decisions in breeding programs, chromosomes or other portions of the genome that are not sufficiently represented by the low-density genotyping platforms used to impute high-density genotypes, will be subject to less or no selection emphasis. Therefore, imputation algorithms should be able to impute genotypes for all chromosomes or regions. Ignoring the sex chromosomes in avian species may affect selection decisions, because chromosome Z, unlike sex chromosomes in mammals, is important in poultry due to its relatively long length; it contains more than 730 genes (Chicken genome assembly 2, ENSEMBL release 64) and accounts for approximately 8% of the total physical genome. Chromosome Z is expected to harbor genetic variation relevant to commercial breeding programs and consequently should have selection emphasis placed upon it.

The objective of this research was to adapt the imputation algorithm used in AlphaImpute to enable imputation of genotypes for sex chromosomes in birds and mammals. The performance of the algorithm was evaluated using genotype data on chromosome Z in a commercial chicken population and this was compared to the imputation of genotypes for autosomal chromosomes in the same population.

## Chromosome Z inheritance

Chicken data were used in this study. The inheritance of sex chromosomes in the chicken involves the ZW system. Males are ZZ (homogametic) and females are ZW (heterogametic). Males receive one copy of Z from each of their parents, while females receive one copy of Z only from their male parent and none from their female parent. Chromosome Z has a small pseudo-autosomal region that recombines with chromosome W [5], which can be treated as an autosomal chromosome in AlphaImpute.

## Imputation algorithm

AlphaImpute has four primary components: (1) simple phasing rules; (2) long-range phasing and haplotype library imputation; (3) segregation analysis; and (4) simple genotype imputation rules. These have been extensively described in Hickey et al. [1]. To address the issue of sex chromosome inheritance, some of these components needed to be modified. First, the problem was simplified by artificially doubling the number of gametes carried by the heterogametic gender, so that they were homozygous at all loci for which they were genotyped, which is consistent with standard genotype calling for sex chromosomes by genotyping providers. This also means that heterogametic individuals are phased *de-facto* for any SNP at which they are genotyped. The simple inheritance based phasing and genotype imputation rules were modified to account for sex chromosome inheritance, i.e. heterogametic individuals inherit alleles only from the homogametic parent, and the alleles inherited from the heterogametic parent are imputed *de-facto* for any allele the heterogametic parent is genotyped for. For autosomal chromosomes, the long-range phasing component of AlphaImpute uses pedigree information to partition surrogate parents into paternal and maternal groups and also uses simple phasing rules based on pedigree information. However, this partitioning can also be carried out using a pedigree-free approach [3], which is suitable for the inheritance of sex chromosomes. The haplotype library phasing and imputation steps are independent of the mode of inheritance and therefore did not need to be modified, other than to ensure that phasing was performed in the pedigree-free mode. For autosomes, AlphaImpute uses GeneProb [4] to perform segregation analysis for all markers of all animals in the pedigree. However, GeneProb does not account for sex chromosome inheritance. Therefore, AlphaImpute was modified and the segregation analysis was replaced by a step that processes the pedigree downward and passes the average of the parental alleles to each individual. This implies that alleles not fully imputed have numbers that are similar to allele probabilities and consequently have genotypes that are real numbers between 0 and 2 as opposed to integers, 0, 1, or 2, where 0 and 2 are homozygotes and 1 is a heterozygote. The implementation is flexible with regards to the gender of the heterogametic individuals and therefore will work for other species such as cattle, sheep, or pigs. When imputing genotypes on sex chromosomes, a file indicating the gender of each individual in the pedigree must be supplied.

## Data analysis

Performance of the algorithm was assessed using a real chicken dataset from a commercial breeding program, which had a pattern of linkage disequilibrium similar to that described in Andreescu [6]. High-density genotypes for all 1255 individuals from a pedigree of four generations (Gen1, Gen2, Gen3, Gen4) were available for chromosome Z, and for autosomes 2 and 4. Four alternative genotyping scenarios were generated. In each scenario, 164 individuals from 68 half-sib families from the most recent generation were used as the testing set, which correspond to selection candidates whose imputed genotypes would ordinarily be used to calculate their genomic estimate breeding values. The testing set was genotyped using both high-density and low-density genotyping platforms. The high-density platform used was a custom Illumina Infinium array, which consisted of 36 455 SNP of which 1137, 3913, and 2211 were segregating SNP located on chromosomes Z, 2, and 4, respectively in the datasets studied (Table 1). The low-density platform used was the KASPar kbioscience array, which consisted of 384 SNP segregating in the line used in this study. From the 384 SNP of the panel, 25, 41, and 23 were located on chromosomes Z, 2, and 4, respectively.

**Table 1:** Accuracy of imputation (±SD) in the validation animals, number of SNP that were imputed per chromosome, and number of high-density genotyped animals in the training population for genotyping scenarios SC1 to SC4

**Scenario**	**Chromosome Z**		**Chromosome 2**		**Chromosome 4**		**Nb HD**
**Nb SNP**	**1137**		**3913**		**2211**		
	**Acc.**	**Nb SNP edited**	**Acc.**	**Nb SNP edited**	**Acc.**	**Nb SNP edited**	
SC1	0.96 ± 0.06	1083	0.98 ± 0.01	3669	0.98 ± 0.01	2061	1091
SC2	0.93 ± 0.08	1072	0.95 ± 0.02	3638	0.96 ± 0.02	2044	776
SC3	0.89 ± 0.10	1072	0.92 ± 0.08	3649	0.93 ± 0.08	2054	763
SC4	0.91 ± 0.22	749	0. 96 ± 0.02	3774	0. 96 ± 0.02	2192	1438

Nb SNP = number of SNP that were imputed per chromosome; Nb HD = number of high-density genotyped animals in the training population; Acc. = mean accuracy of imputation; Nb SNP edited = number of SNP that survive the internal editing criteria of AlphaImpute; SD = standard deviation of accuracy of imputation.

In scenario 1 (SC1) all individuals in generations 1, 2, and 3 were genotyped at high-density (i.e. the parents, grand-parents and great grand-parents of the test individuals and a number of other individuals spread across these three generations who were not ancestors of the test individuals) and only the test candidates (generation 4) were genotyped at low-density. Scenario 2 (SC2) was the same as SC1, except that the female ancestors of the test individuals were genotyped at low-density. In scenario 3 (SC3), all individuals in generation 1 were genotyped at high-density, while all ancestors in generations 2 and 3, and the test candidates were genotyped at low-density. In scenario 4 (SC4), the algorithm was further evaluated in a larger dataset, consisting of seven generations, where the first three were the same as in SC1 but in the subsequent three generations only the males were genotyped with the high-density panel, while female ancestors and individuals of both sexes in testing generation 7 were genotyped with the low-density panel. Thus, SC4 was an extension of SC2, with more generations separating high-density female ancestors and test individuals.

Imputation accuracy was assessed as the correlation between true and imputed genotypes [7]. Unlike other measures of imputation accuracy, this statistic accounts for the effect of allele frequency on imputation accuracy [8] and it allows for the evaluation of markers that are imputed as real numbers between 0 and 2 (i.e. dosage) rather than as genotypes coded as integers (0/1/2) [1]. AlphaImpute does not impute all markers as integer genotypes but rather supplies genotype probabilities for those that do not have full information for imputation. For example, markers in the region between two informative markers that surround a detected recombination location cannot have their genotypes imputed with certainty. In these regions AlphaImpute first detects a recombination event, then it finds the nearest informative marker on either side of the recombination location. The distance between these two markers is used as a weight to determine the emphasis given to the alleles on each of the parental gametes in the imputed genotype, which results in an imputed genotype that is not an integer.

## Results

The accuracy of imputation was high for all scenarios and for all chromosomes, although the low-density panel had only a density equivalent to 384 markers across the whole genome (Table 1), which is approximately one SNP every 8 to 9 centimorgans. The accuracy of imputation was slightly lower for chromosome Z than for the two autosomes. Both the accuracy and the differences in accuracy between chromosome Z and the two autosomes were affected by the genotyping status of the immediate ancestors of the test individuals.

Scenario SC1, which had all ancestors genotyped at high-density, had a higher accuracy of imputation than SC2, which had only male ancestors at high-density and female ancestors at low-density, and than SC3, which had only great-grandparents genotyped at high-density and all other ancestors at low-density. Scenario SC4 was a more extreme case of SC2, in which the test individuals were three additional generations removed from their female ancestors that were genotyped at high-density. Despite this, the accuracy of imputation did not appear to be worse for the autosomes in SC4 compared to SC2, but it was slightly lower in SC4 for chromosome Z (still within the bounds of sampling error due to SC4 having a large sampling variance). The genotyping status of the immediate ancestors of the testing individuals has been shown to be an important factor in determining imputation accuracy for autosomal chromosomes in other species, e.g. [1,8]. In this study, this trend was also observed for chromosome Z.

The accuracy of imputation on chromosome Z was much more variable across individuals than it was for the two autosomes. With the exception of SC3, for which it was 0.08, the standard deviation of accuracy was at most 0.02 for the autosomes. For chromosome Z, the variability was large and increased with the increasing difficulty of the imputation scenario. For SC3 and SC4, the standard deviations of accuracy were 0.10 and 0.22 respectively. Thus, although the mean accuracy was lower for chromosome Z than for the autosomes, some individuals had high accuracy, while others had low accuracy. The low accuracy in certain individuals for chromosome Z was due to the higher rate of Mendelian errors for chromosome Z in comparison to the autosomes, which in turn may be caused by lower reliability of genotyping platforms for markers on sex chromosomes than for autosomes. AlphaImpute checks for consistency between the genotype information and the pedigree. Individual SNP genotypes are set to missing in both the parent and the offspring if they conflict. This results in removal of SNP that exceed a threshold for the proportion of individuals having that SNP missing from the full imputation involving the use of haplotype information. For autosomes, these SNP are imputed using single-locus segregation analysis [4] but for sex chromosomes they are naively imputed as the parent average genotype. For chromosome Z, particularly for SC4, a greater proportion of SNP were excluded from the analysis than for the autosomes (Table 1).

The good performance of imputation of genotypes on chromosome Z for some individuals can be explained by the fact that imputation of markers on sex chromosomes is less challenging than on autosomes for a number of reasons. Heterogametic individuals are phased *de-facto*, thus avoiding the possibility of phasing errors for these individuals, other than due to genotyping errors. The highly accurate phasing of heterogametic individuals helps in surrogate definition and partitioning in the long-range phasing step, and in the haplotype library phasing step of AlphaImpute for homogametic individuals. Imputation of the gamete received from the heterogametic parent by a homogametic individual is also *de-facto*. Computation time for imputation for all chromosomes was of the order of minutes for this dataset but was faster for chromosome Z than for the autosomes, because the phasing was computationally less demanding and genotype probabilities were not calculated for the reasons aforementioned.

Using the imputation approach outlined in this paper, which was specifically designed to impute genotypes on sex chromosomes, did not always outperform the standard autosomal imputation procedure of AlphaImpute. Using the autosomal approach yielded imputation accuracies of 0.97 ± 0.04, 0.92 ± 0.08, 0.84 ± 0.11, and 0.89 ± 0.04 for SC1, SC2, SC3, and SC4, respectively. The autosomal approach was better than the specifically designed approach for SC1 but worse for the three remaining scenarios. Good performance of the autosomal approach for imputation of sex chromosomes may be due to the pedigree haplotype library imputation step, which is independent of the mode of inheritance. However, in the presence of highly accurate genotyping of sex chromosome markers and high-density genotypes on close ancestors of the individuals to be imputed, the imputation approach outlined in this paper is expected to be more optimal than the standard autosomal imputation approach implemented in AlphaImpute.

The pseudo-autosomal region of chromosome Z and chromosome W was ignored in this study due to the difficulty in both identifying and sequencing SNP in this region. If these can be reliably identified, they can be treated as an artificial autosomal chromosome in AlphaImpute. Compared to chromosome Z, chromosome W is very small, contains only a handful of known genes [5] and has very few known SNP reported in Assembly 2 of the chicken genome.

## Conclusions

AlphaImpute was modified to impute genotypes on sex chromosomes. The high accuracy of imputation for chromosome Z obtained in this study, coupled with the previously obtained high accuracy of imputation for autosomes, makes routine implementation of low-cost genomic selection in chickens possible for most of the genome. AlphaImpute is freely available for research purposes from http://sites.google.com/site/hickeyjohn.

## Competing interests

The authors declare that they have no competing interests.

## Authors’ contributions

JMH and AK conceived the experiment, wrote the code, analysed the data, and wrote the paper. Both authors read and approved the final manuscript.
